# Preoperative serum hepatitis B virus DNA was a risk factor for hepatocellular carcinoma recurrence after liver transplantation

**DOI:** 10.1080/07853890.2022.2107233

**Published:** 2022-08-05

**Authors:** Dali Zhang, Danni Feng, Minjuan Ren, Ying Bai, Zhenwen Liu, Hongbo Wang

**Affiliations:** Senior Department of Hepatology, The Fifth Medical Center of Chinese People's Liberation Army General Hospital, Beijing, China

**Keywords:** Liver transplantation, hepatocellular carcinoma, hepatitis B virus DNA, recurrence, long-term survival

## Abstract

**Background:**

Tumour characteristics and orthotopic liver transplantation (OLT) criteria are risks for recurrence of hepatocellular carcinoma (HCC). In Asia, most HCC is caused by chronic hepatitis B infection. Whether hepatitis B virus DNA (HBV DNA) is a risk factor for HCC recurrence after OLT is not clear.

**Patients and methods:**

In this retrospective study, we classified patients into groups of detectable and undetectable HBV DNA, non-HCC recurrence, and recurrence and performed analyses on differed characteristics between groups and risk factors for HCC recurrence after OLT.

**Results:**

Among patients who underwent OLT for HCC, 117 were secondary to CHB infection. CHB was not a risk, but advanced tumour characteristics were risk factor for HCC recurrence. In patients with CHB-HCC, 24 (20.5%) of 117 patients had HCC recurrence. Compared to patients with HBV DNA undetectable (*n* = 75), patients with detectable HBV DNA (*n* = 42) had higher AFP concentration (*p* < .001), higher proportion of macrovascular invasion (*p* = .014), greater tumour diameter (*p* < .001), poorer TNM stage (*p* = .017), and higher proportion of extended OLT criteria (*p* = .011) and HCC recurrence (*p* = .036). Preoperative HBV DNA >2000 IU/mL was an independent risk factor for HCC recurrence (OR = 8.35, 95% CI 1.40, 50.00, *p* = .020). HBV DNA detectable was not a risk for HCC-related death.

**Conclusion:**

Individuals with preoperative undetectable HBV DNA had advanced tumour characteristics and a higher proportion of HCC recurrence. Antiviral treatment for HCC should be performed, and HBV DNA undetectable should be obtained before OLT. But for an urgent OLT, preoperative detectable HBV DNA may not affect long-term survival.

**KEY MESSAGES**
- Patients with HBV DNA detectable had advanced tumour characteristics, a higher proportion of extended OLT criteria, and HCC-recurrence.
- HBV DNA >2000 IU/mL was a risk factor for HCC recurrence.
- HBV DNA detectable was not a risk for HCC related death; extended OLT criteria affected long-term survival.

## 1. Background

Hepatocellular carcinoma (HCC) is the most common liver cancer, accounting for 90% of all liver cancer cases [1]. Chronic hepatitis B infection (CHB) is the leading cause of HCC and liver-related death in Asia.

Orthotopic liver transplantation (OLT) is a curative procedure for patients with HCC who meet Milan criteria. Even so, HCC might be recurrent. Tumour characteristics, such as alpha-fetoprotein (AFP) concentration, tumour diameter, macrovascular invasion, and extended OLT criteria, are established risk factors for HCC recurrence [2,3]. In Asia, the most common aetiology of HCC is CHB infection. A serum hepatitis B virus DNA (HBV DNA) level is correlated with the risk of HCC [2,4,5], and serum HBV DNA detectable and HBV DNA >2000 IU/mL are risk factors for HCC recurrence after hepatectomy [4,6,7]. Whether preoperative HBV DNA status is a risk for HCC recurrence after OLT is unclear, therefore, we recruited patients with HCC who had OLT from a referral medical centre and explored risk factors for HCC recurrence and characteristics of patients with HCC and detectable HBV DNA.

## 2. Methods

### 2.1. Study design

We conducted a retrospective study of patients with HCC who had OLT at the fifth medical centre of Chinese people’s liberation army general hospital from January 2015 to February 2019 and collected data from the electronic medical records system of this hospital. Patients were grouped into preoperative detectable and undetectable HBV DNA, post-OLT HCC recurrence, and non-recurrence. The liver transplantations performed in this study were all deceased classic OLT. All organs were from donors. This study protocol conformed to the ethical guidelines of the 1975 Declaration of Helsinki.

### 2.2. Study population

All patients with HCC who underwent liver transplantation met the following inclusion criteria:

1. Consecutive patients underwent liver transplantation in our hospital from January 2015 to February 2019.
2. Diagnosis with HCC based on the liver pathology, imaging, and AFP concentration.
3. Age 18-70 years.

Patients were excluded who met the following criteria:

1. Combined with other organ transplantation.
2. Cholangiocarcinoma or combined with other malignant tumours.
3. Incomplete follow-up data.

### 2.3. HCC diagnosis

All patients were diagnosed with HCC before OLT. The HCC diagnosis was based on liver pathology, imaging, and AFP. After OLT, diagnosis with HCC recurrence was following the Barcelona Clinic Liver Cancer diagnostic criteria [2].

### 2.4. The status of HBV DNA

Undetectable HBV DNA was defined as <20 IU/mL by a sensitive polymerase chain reaction assay, and detectable HBV DNA was more than 20 IU/mL.

### 2.5. Liver transplant criteria for HCC

Most of the patients in our study met the following OLT criteria.

Milan criteria: (1) single tumour diameter ≤ 5 cm, (2) multiple tumour nodules ≤ 3, and each nodule ≤ 3 cm, and (3) no vessels invasion and no metastases.

Up-to-seven criteria: a number of tumours plus the total tumour diameter ≤ 7, no vessel invasion, and no metastases.

Hangzhou criteria: (1) no major vascular invasion or extrahepatic metastasis, (2) total diameter of tumours ≤8 cm or >8 cm with preoperative AFP ≤ 400 ng/mL, and histological studies with high or moderate differentiation.

### 2.6. Immunosuppression regimen

All patients received tacrolimus, mycophenolate mofetil, and prednisone after liver transplantation. Prednisone was tapered and terminated one month after liver transplantation.

### 2.7. Data collection

We screened the medical records in our hospital system. Data included demographic information, HBV DNA status, aetiology, and tumour characteristics such as tumour size, number, total tumour diameter, preoperative AFP, and vascular invasion. We obtained pre-OLT tumour size, number, total tumour diameter, and microvascular invasion based on pathology.

### 2.8. Statistics

Continuous variables with normal distribution were reported as the mean and standard deviation, and non-normal distributions were reported as the median and interquartile range. Categorical variables were expressed as a percent. T-test and Mann-Whitney test were used for comparing means or medians, and the Chi-squared test was used for comparing categorical variables. Logistic regression models were conducted to obtain the association between detectable HBV DNA, OLT criteria, and recurrence. A Kaplan-Meier analysis was used to assess overall survival (OS) and disease-free survival (DFS), and a log-rank test was used to compare patient survival according to various transplantation criteria. A *p*-value <.05 was considered to represent significance. IBM SPSS 25 was used in this study.

## 3. Results

### 3.1. Characteristics of patients with HCC before liver transplantation

Our hospital electronic medical records system identified 170 patients who underwent OLT for HCC from January 2015 to February 2019. After the medical record review, we excluded four patients who had cholangiocarcinoma. One hundred and sixty-six patients were recruited; 117 of them presented with chronic hepatitis B infection, which accounted for 70.5% (*n* = 117). There were 146 (88.0%) men, with a median age of 52.77 ± 0.65 years, and 20 (12.0%) women, with a median age of 55.90 ± 2.76 years. The median maximal tumour diameter was 3.0 (1.8, 5.0) cm; the median tumour count was 1 (1, 2), and the median AFP concentration was 9.9 (3.7, 88.6) ng/mL. Of these patients with HCC, 60.8% had microvascular invasion, and 15.1% had a macrovascular invasion.

### 3.2. Preoperative tumour characteristics were risk factors for HCC recurrence, not CHB infection

**Figure 1.**
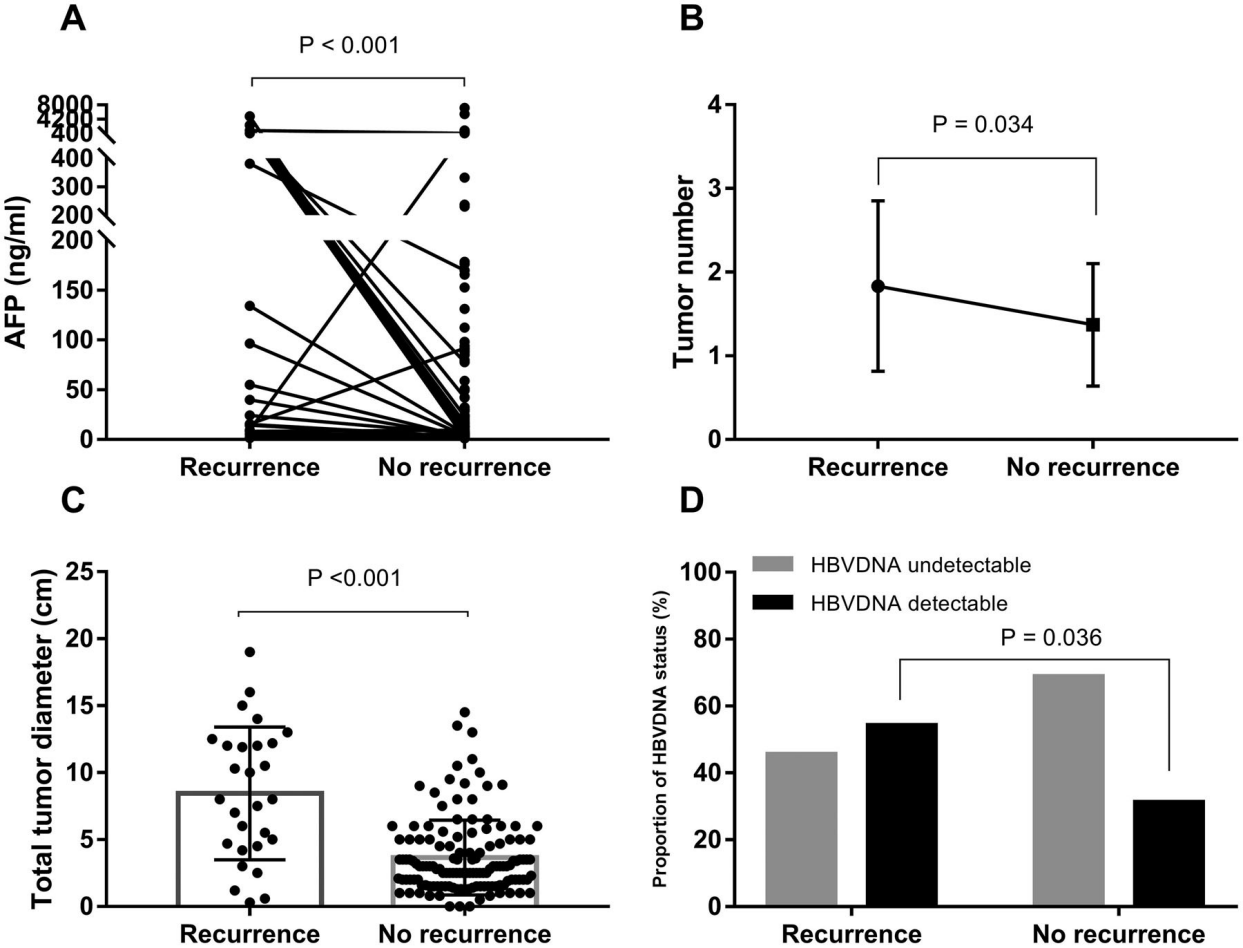
Patients with HCC recurrence had advanced tumour characteristics and a higher proportion of preoperative HBV DNA detectable than those with no recurrence. (A) Patients with HCC recurrence had higher AFP concentrations than patients who did not experience recurrence (*p*<.001). (B) Patients with HCC recurrence had greater tumour numbers compared with patients who did not experience recurrence (*p*=.034). (C) Patients with HCC recurrence had a longer total tumour diameter than patients without recurrence (*p*<.001). (D) In patients with HCC recurrence, 54.2% (*n* = 13) patients had preoperative detectable HBV DNA and 45.8% (*n* = 11) had undetectable HBV DNA. Patients with HCC recurrence had a higher proportion of preoperative detectable HBV DNA than patients without recurrence (*p*=.036).

During the median of 43 months of follow-up, 31 out of 166 (18.67%) patients had HCC recurrence. These patients with HCC were classified into HCC recurrence and non-recurrence after OLT. Patients with HCC recurrence had larger maximal tumour size and a greater tumour count, higher AFP concentration (Figure 1), poorer TNM stage (*p* = .001), and higher prevalence of vascular invasion (*p* < .001) than those with non-recurrence. Preoperative CHB infection was not a risk factor for HCC recurrence after OLT.

#### 3.2.1. Preoperative serum HBV DNA was a risk factor for HCC recurrence in patients with CHB-HCC

CHB infection is a risk factor for HCC but not a risk for HCC recurrence after OLT. HBV DNA detectable and HBV DNA >2000 IU/mL are risk factors for HCC recurrence after liver resection. Whether they are risks for HCC recurrence after OLT is unclear. Thus, we conducted a subgroup analysis of 117 patients who had HCC secondary to CHB infection.

Among these 117 patients with CHB-HCC; 42 (35.9%) patients presented with HBV DNA detectable, 13 (11.1%) patients with HBV DNA >2000 IU/mL. During 43 months follow-up, 24 (20.5%) patients had HCC recurrence. These patients with CHB-HCC were grouped into HCC recurrence (*n* = 24) and non-recurrence (*n* = 93) after OLT. Variables with *p*>.1 *via* univariate analysis were included in the multivariate analysis. HBV DNA >2000 IU/mL versus HBV DNA detectable, AFP more than 200 ng/mL versus AFP level had significant means in the multivariate analysis of risk for HCC recurrence. Thus, HBV DNA >2000 IU/mL (*p* = .004), BMI (*p* = .023), AFP more than 200 ng/mL (*p* < .001), tumour counts (*p* = .050), maximal tumour diameter (*p*< .001), macrovascular invasion (*p* < .001), microvascular invasion (*p* = .014), TNM stage (*p* = .004), and MELD score (*p* = .086) were included in the multivariate analysis.

Multivariate analysis showed that independent factors for HCC recurrence were maximal tumour diameter (OR = 1.38, 95% CI 1.09, 1.75, *p* = .009), HBV DNA >2000 IU/mL (OR = 8.35, 95% CI 1.40, 50.00, *p* = .020), and macrovascular invasion (OR = 11.35, 95% CI 2.25, 57.24, *p* = .003).

#### 3.2.2. Patients with detectable HBV DNA had advanced tumour characteristics before liver transplantation

Tumour characteristics are risk factors for HCC recurrence, and patients with detectable HBV DNA had a significantly higher proportion of HCC recurrence (*p* = .036). Thus, we conducted subgroup studies to explore tumour characteristics between patients with detectable (*n* = 42) and undetectable (*n* = 75) HBV DNA in the CHB-HCC setting.

**Figure 2.**
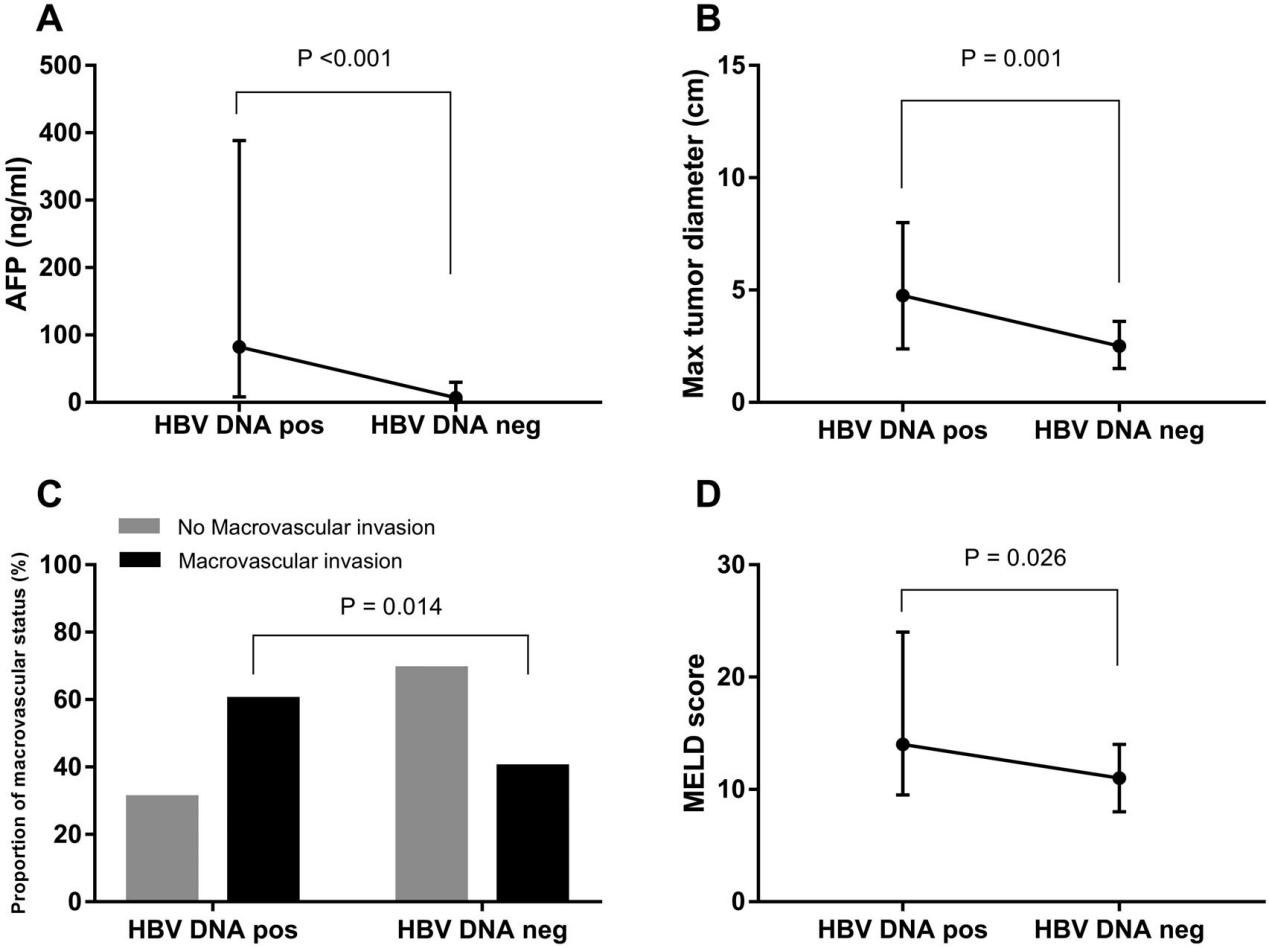
Patients with detectable HBV DNA before liver transplantation had advanced tumour characteristics. (A) Patients with preoperative detectable HBV DNA had higher AFP concentrations compared to those with undetectable HBV DNA (*p*<.001). (B) Patients with preoperative detectable HBV DNA had longer maximal tumour diameter compared to those with undetectable (*p*<.001). (C) In patients with macrovascular invasion, 60% (*n* = 12) of them had preoperative detectable HBV DNA, and 40% (*n* = 8) was preoperative undetectable HBV DNA. Patients with macrovascular invasion had a higher proportion of preoperative detectable HBV DNA (*p* = .014). (D) Patients with preoperative detectable HBV DNA had a higher MELD score than those with undetectable (*p*=.026). HBV DNA pos: HBV DNA detectable; HBV DNA neg: HBV DNA undetectable.

Among these 117 patients with CHB-HCC, patients with detectable HBV DNA had higher AFP concentration (*p* < .001), longer total (*p* < .001) and maximal (*p* = .001) tumour diameter, poorer TNM stage (*p* = .017), a higher percent of macrovascular invasion (*p* = .014) and microvascular invasion (*p* = .029), and higher MELD (*p* = .026) and Child scores (*p* = .039) than those with undetectable HBV DNA (Figure 2 and Table 1). Patients with detectable HBV DNA had advanced tumour characteristics and had a higher prevalence of HCC recurrence than those with undetectable HBV DNA.

**Table 1.** Patients with detectable HBV DNA before liver transplantation had worse tumour characteristics.

	Groups		
Variables	HBV DNA neg (*n* = 75)	HBV DNA pos (*n* = 42)	*p*
Recurrence			.036
0	64 (85.3%)	29 (69.0%)	
1	11 (14.7%)	13 (31.0%)	
BMI (Kg/m^2^)	24.2(22.4, 26.1)	24.7(22.5, 28.1)	.264
AFP (ng/mL)	6.7(3.0,29.4)	82.3(8.3, 382.2)	<.001
Tumour counts	1 (1, 2)	1 (1, 3)	.166
Max tumour diameter (cm)	2.5(1.5, 3.6)	4.8 (2.5, 8.0)	.001
Macrovascular invasion	8 (10.7%)	12 (28.6%)	.014
Microvascular invasion	46 (61.3%)	34 (81.0%)	.029
TNM stage			<.001
I + II	78(83.9%)	12(50.0%)	
III + IV	15(16.1%)	12(50.0%)	
MELD score	11.0 (8.0, 14.0)	14.0 (9.0, 24.0)	.026
Child score	9.0 (8.0, 10.0)	10.0 (8.0, 11.0)	.039

HBV DNA neg: HBV DNA undetectable; HBV DNA pos: HBV DNA detectable; BMI: Body mass index; AFP: alpha-fetoprotein; MELD: model for end stage liver disease.

#### 3.2.3. Patients with detectable HBV DNA before liver transplantation had a higher proportion of extended OLT criteria

Milan criteria are landmarks for OLT. The extended criteria like “Up-to-seven” and Hangzhou criteria are proposed for benefiting more patients from OLT but might have higher risks of HCC recurrence and poorer long-term survival. We performed the subgroups analysis to figure out whether patients with detectable HBV DNA had a higher proportion of extended OLT criteria than those with undetectable HBV DNA and whether patients with extended OLT criteria had higher risks for HCC recurrence than Milan criteria.

**Figure 3.**
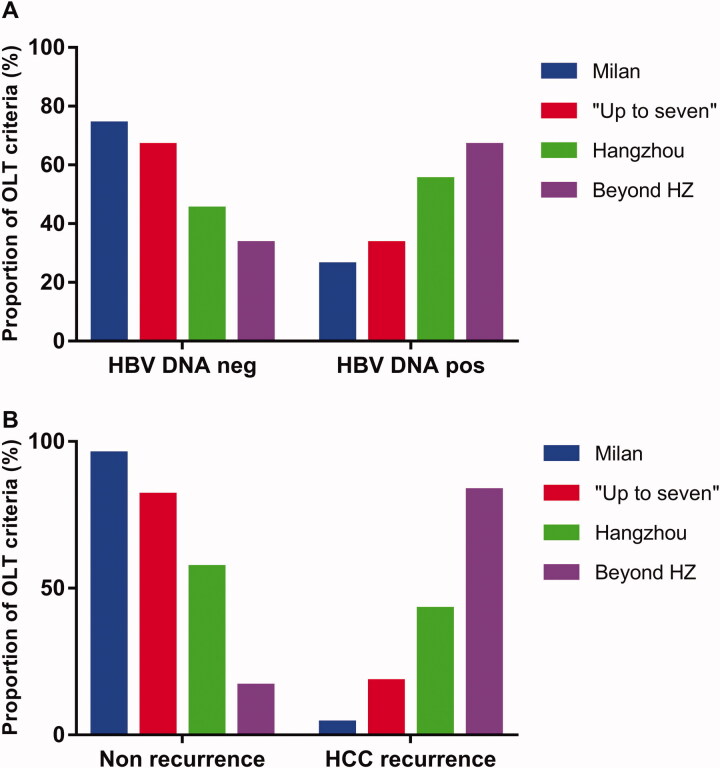
Patients with detectable HBV DNA before liver transplantation had a higher prevalence of extended OLT criteria, and extended OLT criteria had a higher prevalence of recurrence than Milan criteria. (A) There were 26% (*n* = 19), 33.3% (*n* = 4), 55.0% (*n* = 11), and 66.7% (*n* = 8) preoperative detectable HBV DNA patients in Milan, beyond Milan within “Up-to-seven” out of “Up-to-seven” within Hangzhou, and beyond Hangzhou criteria, respectively (*p*=.011). (B) The prevalence of HCC recurrence was 4.1% (*n* = 3), 18.2% (*n* = 2), 42.9% (*n* = 9), and 83.3% (*n* = 10) that was significantly different between patients with Milan criteria, beyond Milan and within “Up-to-seven” beyond “Up-to-seven” and within Hangzhou, and beyond Hangzhou criteria, respectively (*p*<.001). *Beyond HZ: Beyond Hangzhou criteria. HBV DNA pos: HBV DNA detectable; HBV DNA neg: HBV DNA undetectable.

Patients with detectable HBV DNA had a higher proportion of extended OLT criteria (54.8%) than those with undetectable (28.0%; *p* = .011), which was consistent with the results that patients with detectable HBV DNA had advanced tumour characteristics than those with undetectable (Figure 3(A)).

Patients with extended OLT criteria had higher risks for HCC recurrence than Milan criteria. The prevalence of HCC recurrence was 4.1% (3/73), 18.2% (2/12), 42.9% (9/20), and 83.3% (10/12) in patients with Milan criteria (*n* = 73), beyond Milan and within “Up-to-seven” (*n* = 12), beyond “Up-to-seven” and within Hangzhou (*n* = 20), and beyond Hangzhou criteria (*n* = 12), respectively (*p*<.001). These results showed that extended OLT criteria had a statistically higher prevalence of HCC recurrence (Figure 3(B)).

#### 3.2.4. Extended OLT criteria affected long-term survival, not detectable HBV DNA

Patients with detectable HBV DNA had a higher proportion of extended OLT criteria than those with undetectable. However detectable HBV DNA was not a risk for HCC-related death. For these 117 recipients with CHB-HCC, the 4-year OS after liver transplantation was 85.3%; DFS was 75.2%. Among these recipients, 14 (12.0%) patients had HCC-related death. Multivariate analysis showed that preoperative serum detectable HBV DNA was not a risk factor for HCC-related death (OR = 2.71, 95% CI 0.87–8.42). The independent factors for death were AFP more than 200 ng/mL (OR = 2.70, 95% CI 1.33–5.50) and macrovascular invasion (OR = 12.57, 95% CI 3.25–48.62).

**Figure 4.**
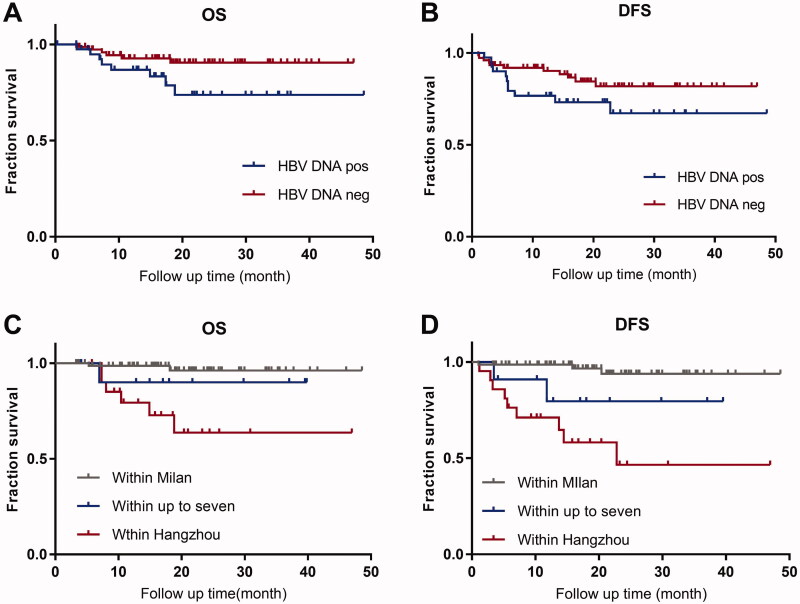
Survival of patients with HCC with different HBV DNA status and liver transplant criteria. Patients with detectable HBV DNA did not have any differences in OS (*p*=.056; Figure 4(A)) and DFS (*p*=.094; Figure 4(B)) compared with those with undetectable. Compared with Milan criteria, patients beyond Milan within the “Up-to-seven” criteria had similar outcomes in OS (*p*=.27; Figure 4(A)) and DFS (*p*=.06; Figure 4(B)); patients who met Hangzhou criteria had significant differences in OS (Figure 4(C)) and DFS (Figure 4(D); *p*< .001). HBV DNA pos: HBV DNA detectable; HBV DNA neg: HBV DNA undetectable.

Patients with detectable HBV DNA did not affect long-term survival. Patients with preoperative serum detectable HBV DNA had no differences in OS (*p* = .056) and DFS (*p* = .094) than those with undetectable (Figure 4(A,B)) after OLT.

Patients with extended OLT criteria affected long-term survival. Patients with different OLT criteria had a significant means in OS (*p* < .001) and DFS (*p* < .001; Figure 4(C,D)). The OS was 96.1%, 90.0%, and 63.6%, respectively, in patients within Milan criteria, beyond Milan and within “Up-to-seven,” and beyond “Up-to-seven” and within Hangzhou; and similarly, the DFS was 93.8%, 79.5%, and 46.5% respectively.

## 4. Discussion

CHB infection is a risk for HCC but not a risk for HCC recurrence after OLT. We found that, in 117 patients with CHB-HCC, patients with preoperative serum detectable HBV DNA had higher AFP concentrations, longer tumour diameter, a higher percent of macrovascular invasion, microvascular invasion, and poorer TNM stage, and a higher proportion of extended OLT criteria than those with undetectable HBV DNA. These characteristics were risk factors for HCC recurrence. Furthermore, preoperative serum HBV DNA >2000 IU/mL was an independent risk factor for HCC recurrence after OLT. However, HBV DNA detectable was not a risk factor for HCC -related death and did not affect long-term survival.

Patients with detectable HBV DNA had advanced tumour characteristics and a higher proportion of HCC recurrence. The reason might be detectable HBV DNA per se promote tumour cell growth. Studies reported that preoperative serum HBV DNA and Ishak hepatic inflammation score are risk factors for HCC recurrence after hepatectomy [4,6,7]. We found that hepatic inflammation was not a risk factor for HCC recurrence because patients had a normal liver function after OLT. However, serum high HBV DNA load was still a risk factor for HCC recurrence. Possibly, the binding of HBV × protein to nuclear transcription factors promotes tumour cell growth [8], and HBV infection induces liver progenitor cell activation and abnormal genetic changes, resulting in malignant transformation [9]. This possibility might explain why patients with HBV DNA in our study had large tumour sizes, severe tumour invasiveness, and advanced tumours. And this present study suggests that antiviral treatment is important, and HBV DNA undetectable is even better. This is consistent with a randomized clinical trial that showed that nucleotide/nucleoside analog treatments decreased HBV-HCC recurrence [10]. Some studies indicated that serum HBV DNA status is a more sensitive criterion compared with HBV DNA titre in liver tissues [8,11–15]. In this current study, we refer to serum HBV DNA.

Patients with detectable HBV DNA had a higher proportion of extended OLT criteria than those with undetectable and extended liver OLT criteria had a higher prevalence of HCC recurrence. Liver transplantation is the optimal option for patients with HCC who meet Milan criteria. Many extended criteria were proposed to benefit more patients with HCC and are adequate models to predict HCC recurrence and long-term survival [16–21]. The proportion of extended liver OLT criteria was 54.8% and 28.0% in patients with detectable and undetectable HBV DNA, respectively. Patients with extended liver OLT criteria (Up-to-seven, Hangzhou, and beyond Hangzhou criteria) had a higher prevalence of HCC recurrence (18.2%, 42.9%, and 83.3%) than Milan criteria (4.1%).

HBV DNA detectable was not a risk for HCC-related death and did not affect long-term survival, and extended liver OLT criteria affected long-term survival. Preoperative HBV DNA detectable was not a risk for HCC-related death that might be partially due to the use of high genetic barrier nucleos(t)ide analogue(s) and HCC recurrence matter much to long-term survival. AFP and macrovascular invasion, which are risks for death and components defined as extended OLT criteria, affected long-term survival. In this study, patients with detectable HBV DNA had no differences in OS (*p* = .056) and DFS (*p* = .094) than those with undetectable. Extended liver transplant criteria were risk factors for HCC recurrence and long-term survival. The 4-year OSs were 96.1%, 90.0%, 63.6%, and 57.1% in patients who met the Milan criteria, exceeded Milan criteria and met the “Up-to-seven” criteria, exceeded the “Up-to-seven” criteria and met the Hangzhou criteria, and exceeded Hangzhou criteria, respectively (*p*< .001).

A limitation of this study was a moderate sample size, and the study was retrospective. However, we found that patients with detectable HBV DNA had advanced tumour characteristics and higher prevalence of extended OLT criteria, and a higher proportion of HCC recurrence, which suggested that antiviral treatment for HCC should be performed, and undetectable HBV DNA before OLT would be better.

## 5. Conclusions

Patients with preoperative serum detectable HBV DNA had more advanced tumour characteristics and a higher proportion of extended OLT criteria, and HCC recurrence than those with undetectable, and HBV DNA >2000 IU/mL was a risk for HCC recurrence after OLT. Antiviral treatment for HCC should be performed, and undetectable HBV DNA before OLT is necessary. But for an urgent OLT, preoperative HBV DNA detectable did not affect long-term survival; extended OLT criteria were affected.

## Data Availability

We will share de-identified participant data. The exact details of what data and how it will be shared have not been fully determined as some of our research staff were suspended with COVID-19 and have only partially resumed. Please contact Dr Dali Zhang for more details at zhangdali20051019@163.com.

## References

[1] Akinyemiju T, Abera S, Ahmed M, Global Burden of Disease Liver Cancer Collaboration, et al. The burden of primary liver cancer and underlying etiologies from 1990 to 2015 at the global, regional, and national level: Results from the global burden of disease study 2015. JAMA Oncol. 2017;3(12):1683–1691. 28983565 10.1001/jamaoncol.2017.3055 PMC5824275

[2] European association for the study of the liver. Electronic address EEE, European association for the study of the L. EASL clinical practice guidelines: Management of hepatocellular carcinoma. J Hepatol. 2018;69(1):182–236. 29628281 10.1016/j.jhep.2018.03.019

[3] Duffy JP, Vardanian A, Benjamin E, et al. Liver transplantation criteria for hepatocellular carcinoma should be expanded: a 22-year experience with 467 patients at UCLA. Ann Surg. 2007;246(3):502–511. 17717454 10.1097/SLA.0b013e318148c704 PMC1959350

[4] Shen J, Dai J, Zhang Y, et al. Baseline HBV-DNA load plus AST/ALT ratio predicts prognosis of HBV-related hepatocellular carcinoma after hepatectomy: a multicentre study. J Viral Hepat. 2021;28(11):1587–1596. 34464991 10.1111/jvh.13606

[5] Heimbach JK, Kulik LM, Finn RS, et al. AASLD guidelines for the treatment of hepatocellular carcinoma. Hepatology. 2018;67(1):358–380. 28130846 10.1002/hep.29086

[6] Yu SJ, Kim YJ. Hepatitis B viral load affects prognosis of hepatocellular carcinoma. World J Gastroenterol. 2014;20(34):12039–12044. 25232241 10.3748/wjg.v20.i34.12039 PMC4161792

[7] Chun J, Kim W, Kim BG, et al. High viremia, prolonged lamivudine therapy and recurrent hepatocellular carcinoma predict posttransplant hepatitis B recurrence. Am J Transplant. 2010;10(7):1649–1659. 20642687 10.1111/j.1600-6143.2010.03162.x

[8] Lei Z, Xia Y, Si A, et al. Antiviral therapy improves survival in patients with HBV infection and intrahepatic cholangiocarcinoma undergoing liver resection. J Hepatol. 2018;68(4):655–662. 29155069 10.1016/j.jhep.2017.11.015

[9] Zhou YM, Cao L, Li B, et al. Expression of HBx protein in hepatitis B virus-infected intrahepatic cholangiocarcinoma. Hepatobiliary Pancreat Dis Int. 2012;11(5):532–535. 23060400 10.1016/s1499-3872(12)60219-7

[10] Yin J, Li N, Han Y, et al. Effect of antiviral treatment with nucleotide/nucleoside analogs on postoperative prognosis of hepatitis B virus-related hepatocellular carcinoma: a two-stage longitudinal clinical study. J Clin Oncol. 2013;31(29):3647–3655. 24002499 10.1200/JCO.2012.48.5896

[11] Choi JG, Chung YH, Kim JA, et al. High HBV-DNA titer in surrounding liver rather than in hepatocellular carcinoma tissue predisposes to recurrence after curative surgical resection. J Clin Gastroenterol. 2012;46(5):413–419. 22105184 10.1097/MCG.0b013e3182371285

[12] Sha M, Jeong S, Xia Q. Antiviral therapy improves survival in patients with HBV infection and intrahepatic cholangiocarcinoma undergoing liver resection: Novel concerns. J Hepatol. 2018;68(6):1315–1316. 29475065 10.1016/j.jhep.2018.01.039

[13] Chen L, Zhang Q, Chang W, et al. Viral and host inflammation-related factors that can predict the prognosis of hepatocellular carcinoma. Eur J Cancer. 2012;48(13):1977–1987. 22325840 10.1016/j.ejca.2012.01.015

[14] Hung IF, Poon RT, Lai CL, et al. Recurrence of hepatitis B-related hepatocellular carcinoma is associated with high viral load at the time of resection. Am J Gastroenterol. 2008;103(7):1663–1673. 18616655 10.1111/j.1572-0241.2008.01872.x

[15] Wu JC, Huang YH, Chau GY, et al. Risk factors for early and late recurrence in hepatitis B-related hepatocellular carcinoma. J Hepatol. 2009;51(5):890–897. 19747749 10.1016/j.jhep.2009.07.009

[16] Mazzaferro V, Regalia E, Doci R, et al. Liver transplantation for the treatment of small hepatocellular carcinomas in patients with cirrhosis. N Engl J Med. 1996;334(11):693–699. 8594428 10.1056/NEJM199603143341104

[17] Yao FY, Ferrell L, Bass NM, et al. Liver transplantation for hepatocellular carcinoma: expansion of the tumor size limits does not adversely impact survival. Hepatology. 2001;33(6):1394–1403. 11391528 10.1053/jhep.2001.24563

[18] Zheng SS, Xu X, Wu J, et al. Liver transplantation for hepatocellular carcinoma: Hangzhou experiences. Transplantation. 2008;85(12):1726–1732. 18580463 10.1097/TP.0b013e31816b67e4

[19] Mazzaferro V, Llovet JM, Miceli R, et al. Predicting survival after liver transplantation in patients with hepatocellular carcinoma beyond the milan criteria: a retrospective, exploratory analysis. Lancet Oncol. 2009;10(1):35–43. 19058754 10.1016/S1470-2045(08)70284-5

[20] Duvoux C, Roudot-Thoraval F, Decaens T, et al. Liver transplantation for hepatocellular carcinoma: a model including alpha-fetoprotein improves the performance of milan criteria. Gastroenterology. 2012;143(4):986–994. 22750200 10.1053/j.gastro.2012.05.052

[21] Mazzaferro V, Sposito C, Zhou J, et al. Metroticket 2.0 model for analysis of competing risks of death after liver transplantation for hepatocellular carcinoma. Gastroenterology. 2018;154(1):128–139. 28989060 10.1053/j.gastro.2017.09.025

